# Integrated Mineral Profiling and Techno-Functional Characterization of Baru (*Dipteryx alata* Vogel) Oil and Almond

**DOI:** 10.1021/acsomega.5c13208

**Published:** 2026-06-04

**Authors:** Pamela F. M. Pereira, Ilma Marques Gomes, Renato Queiroz Assis, Gabriel Bezerra Cardoso, Renata Lázara de Araújo, Danilo Hiroshi Konda, Paula Becker Pertuzatti

**Affiliations:** † Division of Glycoscience, School of Biotechnology, KTH Royal Institute of Technology, AlbaNova University Centre, Stockholm SE−106 91, Sweden; ‡ Institute of Exact and Earth Sciences, 67826 Federal University of Mato Grosso, Barra do Garças 78600-000, Brazil

## Abstract

Baru (*Dipteryx alata* Vogel) is a native Cerrado species with growing nutritional and economic relevance, yet the mineral partitioning between the almond/oil and the resulting techno-functional implications remains unknown. This study provides an integrated characterization of mineral composition, structural features, and physicochemical, rheological, and thermal properties of baru almond and its extracted oil. XRF revealed high levels of essential minerals in the almond, while transition metals showed limited migration into the oil, contributing to its oxidative stability. Conversely, Mg, P, and Zn were proportionally enriched in the oil. The oil exhibited low acidity and peroxide values, a lipid profile dominated by oleic and linoleic acids, Newtonian behavior at processing-relevant shear rates, and high thermal stability (T_onset_ = 394.9 °C). FTIR confirmed the structural integrity of triacylglycerols. Altogether, the selective mineral distribution and balanced lipid composition underpin the chemical and thermal robustness of baru oil, supporting its potential for food and bioeconomic applications.

## Graphical abstract

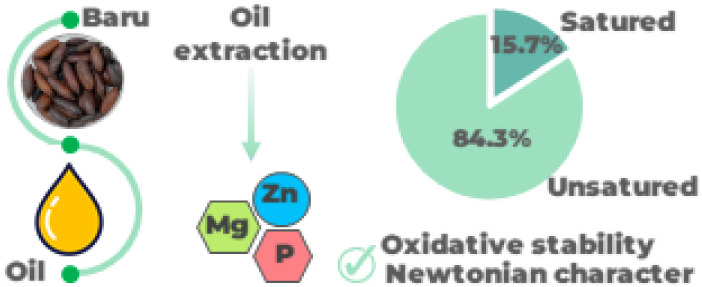

## 1 Introduction

The Brazilian Cerrado is the second largest biome of South America, and its high biodiversity of native species has attracted attention. Among them, baru (*Dipteryx alata* Vogel) almond stands out for its nutritional value and chemical composition.^1^ Consumption of the baru almond has demonstrated numerous health benefits, such as gastrointestinal transit regulation and improvement of biochemical parameters, including triglyceride (TG) and very low-density lipoprotein (VLDL) reduction and high-density lipoprotein (HDL) increase.^2^

In recent years, baru has gained increasing economic relevance within the bioeconomy of the Brazilian Cerrado. In 2025, the market value of baru almond was valued at US$ 420.0 million, with projections reaching US$ 760 million by 2035,^3^ demonstrating the growing commercial interest in this native species and reinforcing the need for studies focused on its composition and technological potential. Regulatory advances have also supported this expansion: since 2021, the Normative Instruction (IN) n° 87 ^4^ has authorized the use of *Dipteryx alata* Vogel (baru) for edible and cosmetic purposes in Brazil. Despite this favorable scenario, most scientific studies concentrate on the composition of the almond and pulp,^5,6^ while the oil remains considerably less explored, especially regarding its physicochemical, thermal, and technological properties. This knowledge gap limits the development of broader industrial applications and highlights the importance of comparative studies involving both matrices.

Baru oil has attracted attention due to its versatility and potential applications in the food, pharmaceutical, and cosmetics industries.^7^ For food processing in particular, understanding properties such as thermal stability, oxidation behavior, and rheology is essential, as they determine formulation performance and processing efficiency.^8^ However, knowledge about baru oil remains limited, and a critical gap concerns its mineral composition. Minerals strongly affect technological properties of vegetable oils, especially oxidation and thermal behavior, and the baru almond is known to contain transition metals such as Mn, Fe, and Cu,^9^ which may be transferred to the oil and affect its stability. Understanding how these minerals are distributed between the almond and the oil is therefore essential to elucidating stability mechanisms and supporting industrial applications.

Given the growing technological interest in baru and the lack of studies on the mineral composition of its oil, we hypothesized that the almond and its extracted oil exhibit distinct mineral profiles that shape their structural, thermal, and functional properties. To test this, we integrated mineral profiling with physicochemical, fatty acid, rheological, and thermal analyses to elucidate the technological potential of *Dipteryx alata*. This study provides the first comparative assessment of mineral distribution between almond and its oil and examines how these compositional patterns relate to techno-functional behavior. By establishing these molecular-functional connections, the work contributes to the valorization of baru within an expanding Cerrado bioeconomy.

## 2 Materials and Methods

### 2.1 Materials

Approximately 5 kg of baru (*Dipteryx alata* Vogel) fruits were collected during the August–September 2020 harvest season in Aragarças, State of Goiás, Brazil (15°53′50″ S, 52°13′48″ W). The collection site is located within the Brazilian Cerrado biome, characterized by a tropical savanna climate with a dry winter season (Aw, Köppen classification), mean annual precipitation around 1,500 mm, and a typical Dystrophic Red-Yellow Latosol soil. The area has an average altitude of approximately 320–400 m above sea level.

Fruits were harvested at full maturity (natural abscission stage) and manually inspected and selected based on integrity, absence of physical damage, and absence of fungal contamination. The almonds were manually extracted using a guillotine-type device fitted with a stainless-steel blade attached to a fixed polyvinyl chloride (PVC) base, ensuring clean and reproducible fracture of the endocarp. The extracted almonds were sanitized and stored in polyethylene bags under refrigeration (4 °C) until oil extraction and subsequent analyses were carried out. All reagents used were of analytical grade.

### 2.2 Oil Extraction Process

The extraction of baru oil was carried out in a Goldfish extractor (Tecnal, TE-044 model, Brazil), using petroleum ether as the solvent. Approximately 13 g of ground almond were placed in the extraction chamber and kept in contact with the solvent under reflux at boiling temperature (95 °C) for 5 h. After extraction, solvent recovery was performed by maintaining the system at 130 °C for 1 h to evaporate the bulk solvent from the reboilers. The reboilers were then removed and transferred to an air-circulating oven (Nova Ética, 400/3 ND model, Brazil) at 105 °C for 30 min to eliminate residual solvent. The extracted oil was added to an amber bottle to protect it from light and stored in a freezer (−18 °C) until further analyses.

### 2.3 Mineral Profile

The mineral composition of baru almond and oil was determined by energy-dispersive X-ray fluorescence spectroscopy (EDXRF) using an Epsilon 4 spectrometer (Malvern Panalytical, Almelo, The Netherlands). The instrument was equipped with a silver-anode X-ray tube operating at 50 kV and an SDD detector under a controlled helium atmosphere to enhance sensitivity for light elements.

For sample preparation, the almond was finely ground, homogenized, and approximately 3 g were transferred to polyethylene sample cups sealed with a 75 μm Mylar film. Liquid oil samples (3 mL) were directly pipetted into identical cups and sealed with the same film. Results were expressed in percentage (%) for major elements and in mg/kg (ppm) for trace elements.

### 2.4 Fourier Transform Infrared Spectroscopy (FT-IR)

The infrared spectrum of baru almond and oil was recorded in a Spectrum 100 spectrometer (PerkinElmer, Waltham, MA, USA) equipped with a Universal Attenuated Total Reflectance (ATR) cell device with a germanium (Ge) crystal. The spectra were obtained with 32 scans at a resolution of 4 cm^–1^ in the wavenumber range of 4000 cm^–1^ to 600 cm^–1^.

### 2.5 Thermogravimetric Analysis (TGA)

The thermal stability of baru almond and oil was evaluated in a thermogravimetric analyzer (Pyris 1 PerkinElmer, Waltham, MA, USA). Thermograms were obtained by heating the samples (3 mg) from 22 to 704 °C under a nitrogen atmosphere and a heating rate of 10 °C/min.

### 2.6 Oil Quality Analysis

For the oil characterization, the acidity index, peroxide index, saponification index, moisture content, and relative density were determined according to the methodology described by Instituto Adolfo Lutz.^10^ The moisture content of oil was determined by the gravimetric method. Relative density was determined by the ratio of the unitary volume weight of the sample and water at 25 °C. All analyses were carried out in triplicates.

### 2.7 Fatty Acids Composition

Fatty acids were converted to methyl esters (FAMEs) according to Hartman and Lago.^11^ Succinctly, 30 mg of oil was subjected to alkaline methanolysis followed by acid esterification. After cooling, FAMEs were extracted with *n*-hexane and analyzed by GC-FID (PerkinElmer Clarus 500) equipped with a Carbowax 20 M column (30 m × 0.25 mm × 0.25 μm). Injector and detector were maintained at 250 °C. The oven program consisted of an initial step at 90 °C, followed by controlled temperature ramps up to 230 °C. FAMEs were identified by comparison with commercial standards (Sigma-Aldrich, St. Louis, USA), and results were expressed as the relative percentage of each fatty acid.

### 2.8 Rheological Characterization

The rheological properties of baru oil were evaluated using a modular rheometer (MCR 102, Anton Paar GmbH, Ostfildern, Germany) equipped with a cone–plate geometry (CP50-1). In all experiments, 750 μL of oil samples and rheological measurements were carried out at different isothermic conditions at 10 °C, 15 °C, 25 °C, 35 °C, and 45 °C. The readings were obtained with permanent control of gap measurements using TruGap in 0.099 mm increments and Toolmaster CP 50; precise temperature control was achieved using T-Ready and Rheoplus V3.61 software. The rheograms were treated with the Rheoplus software. For all apparent viscosity curves, the established parameters were based on shear stress control. The experimental data were fitted to the Newton, Bingham, Ostwald–de Waele (Power law), and Casson models to describe flow behavior, and the respective equations are presented in Supporting Information. The influence of temperature on the consistency and viscosity of baru oil was investigated by the linearization of the Arrhenius equation (eq 1).

1 $$\mu_{\mathrm{ap}}=\eta_{0}e^{E_{a}/RT}$$

where μ_ap_ is the apparent viscosity; η_0_ is the adjustment parameter; *E*_a_ is the activation energy; *R* represents the ideal gas constant; and *T* is the sample temperature.

### 2.9 Statistical Analysis

Data were evaluated for normality using the Shapiro–Wilk test and for homogeneity of variances using the Levene test. When both assumptions were met, one-way ANOVA followed by Tukey’s test (*p* < 0.05) was applied to compare means.

## 3 Results and Discussion

### 3.1 Mineral Profile

The mineral composition of baru almond and oil revealed a complex distribution of macro- and microminerals, with marked differences between matrices and relevant implications for nutritional value, oxidative stability, and techno-functional behavior. Most of the minerals present in baru (Table 1) are considered essential minerals with the exception of Ni, Rb, and Sr. The almond exhibited high levels of Mn, Cu, Fe, Zn, and Ca, which were also shown to have high concentrations in the oil. Based on FDA Daily Values (DV),^12^ 100 g of almond or oil supplies over 100% DV for these minerals. Calcium, iron, and manganese have already been found in high concentrations in the baru pulp, with approximately 30 g of pulp being able to supply the daily recommendation for manganese, and 100 g of pulp containing 10% of the daily recommendation for calcium and 16% of the daily recommendation for iron.^9^ According to Campos,^13^ consuming a 20 g portion of baru almonds per day increased the contribution of iron and calcium by 127% and 6%, respectively, in obese individuals after 60 days of treatment, demonstrating that consuming this almond may improve biochemical and metabolic parameters in humans.

**Table 1:** Mineral Composition of Baru (*Dipteryx alata* Vogel) Almond and Oil and Corresponding Percentage Daily Values (%DV)^a^

Compound	Almond (mg/100 g)	%DV	Oil (mg/100 g)	%DV
Mg	111	26%	270	64%
P	429	34%	825	66%
S	286	-	602	-
K	3861	82%	4296	91%
Ca	1488	115%	1103	85%
Cl	45.0	-	1.0	-
Mn	49.7	2160%	29.8	1296%
Fe	61.3	341%	46.9	260%
Ni	7.3	-	3.5	-
Cu	16.7	1856%	14.9	1656%
Zn	36.8	334%	52.6	478%
Rb	22.6	-	18.3	-
Sr	16.9	-	9.9	-

a %DV according to FDA (2023) and using a 100 g reference serving.

The migration of minerals from the almond to the oil during extraction is not homogeneous, reflecting differences in solubility, chemical form, and molecular interactions among elements. Elements such as Mg, S, P, and particularly Zn exhibited proportionally higher concentrations in the oil fraction, which may be attributed to an enhanced solubility at higher processing temperatures, thereby favoring the mass transfer coefficient. Mitic et al.^14^ demonstrated the different mineral extraction coefficients in aqueous extracts tended to be favored as a function of temperature increase. On the other hand, a secondary hypothesis is that the observed increase in mineral content in the oil may also be associated with the formation of metal-fatty acid complexes, a mechanism previously described for oleic acid-rich matrices. In hazelnuts, it was demonstrated that manganese preferentially associates with oleic acid, forming stable carboxylate-metal structures.^15^ This behavior is favored by the high abundance of oleic acid and by the ability of unsaturated fatty acids to stabilize divalent cations through coordination with the carboxylate group. Considering that baru oil contains more than 70% unsaturated fatty acids (Table 2), the increased levels of Mg and Zn in the oil are chemically plausible and may result, at least in part, from the formation of lipophilic complexes such as magnesium oleate and zinc oleate/linoleate, which are known to partition into the lipid phase during solvent extraction.

**Table 2:** Physicochemical Parameters of Baru Oil^a^

Parameters	Values
Acidity index (mg KOH/g)	0.37 ± 0.13
Saponification index (mg KOH/g)	285.61 ± 2.90
Peroxide index (mg KOH/g)	6.45 ± 0.26
Moisture content (%)	0.31 ± 0.21
Refractive index (n^40^D)	1.48 ± 0.02
Relative density (g/mL)	0.931 ± 0.002

a Mean ± standard deviation of the analyses performed in triplicate.

In contrast, transition metals such as Fe, Cu, and Ni, although detected in relevant amounts, showed markedly lower transfer from the almond to the oil. This reduced migration is consistent with their stronger affinity for hydrophilic cellular structures, protein-bound forms, or chelation by phytates, which are well-documented in baru almonds.^16^ From a technological standpoint, this behavior is noteworthy because these transition metals are well-known catalysts of lipid oxidation, accelerating hydroperoxide decomposition through Fenton- and Haber–Weiss-type reactions.^17,18^ Therefore, their limited partitioning into the oil phase may contribute favorably to oxidative stability, as lower concentrations of catalytic metals reduce the propensity of oils to undergo rapid oxidative deterioration.

### 3.2 FTIR Spectra

The FTIR spectrum of baru oil exhibited the characteristic vibrational profile of triacylglycerol-rich vegetable oils (Figure 1a). The small shoulder at 3008 cm^–1^ confirmed the presence of *cis-*unsaturated fatty acids, while the strong CH_2_ stretching bands at 2920 cm^–1^ and 2843 cm^–1^ reflected long aliphatic chains. A strong and sharp band at 1743 cm^–1^ was assigned to the ester carbonyl stretching (ν­(C=O)) of triacylglycerols and therefore reflects the intact glyceride structure of the oil rather than free fatty acids. A weak signal close to 1650 cm^–1^ is consistent with *cis* C=C stretching of unsaturated acyl chains. Additional bands at 1465 cm^–1^ and 1377 cm^–1^ arise from CH_2_ scissoring and CH_3_ symmetric bending, while the bands at 1155–1110 cm^–1^ correspond to C–O and C–O–C stretching modes of the ester linkage. The absorption at 714 cm^–1^, associated with methylene rocking and out-of-plane bending of *cis* disubstituted olefins, further supports the predominance of oleic and linoleic acids, in agreement with GC results.^19^ The results are similar to the previous study for other vegetable oils.^20^

**Figure 1:**
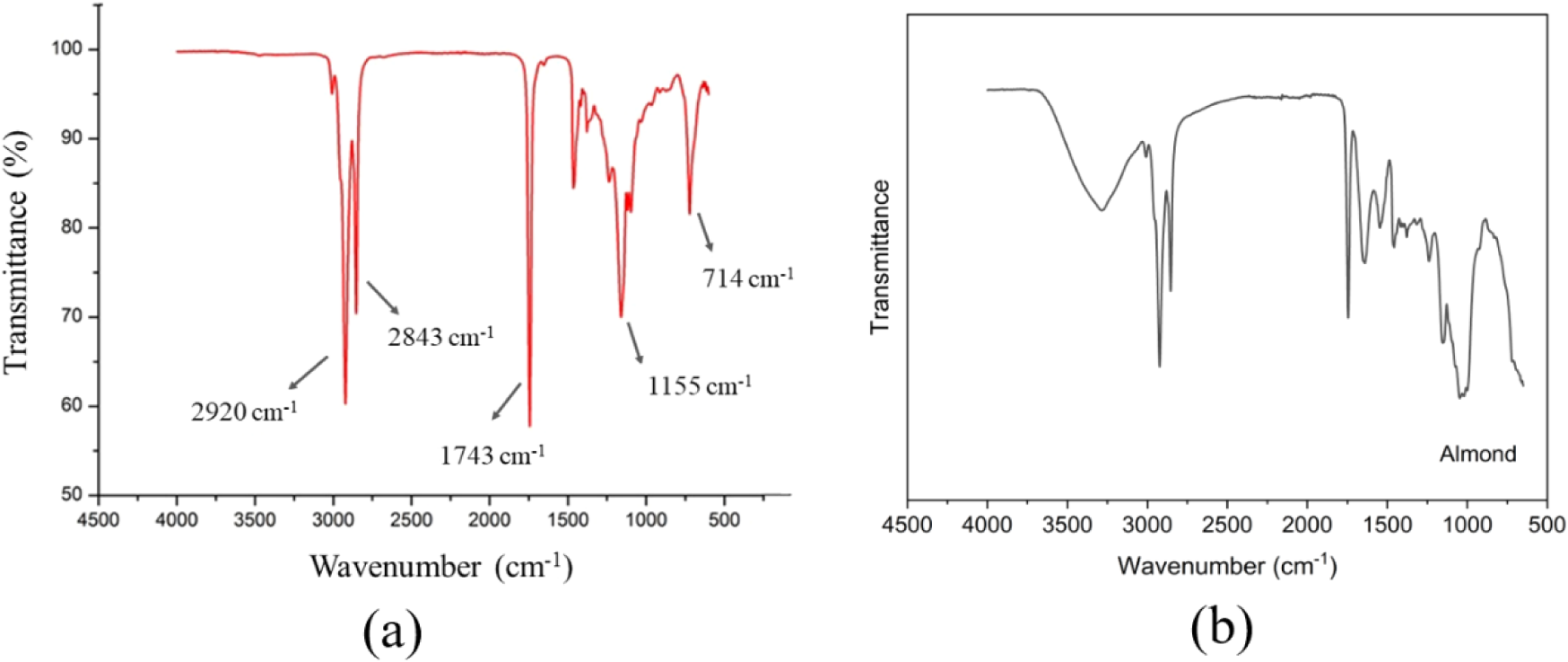
Infrared spectrum of baru oil (a) and almond (b).

In the almond spectrum (Figure 1b), a broader and more complex matrix was observed. The wide band around 3280 cm^–1^ reflected overlapping O–H and N–H stretching from moisture, carbohydrates, and proteins. In addition to the lipid-related CH_2_/CH_3_ bands, the almond showed characteristic protein absorptions at 1640 cm^–1^ consistent with amide I vibrations (C=O stretching of proteins) and 1545 cm^–1^ attributable to amide II, confirming the presence of proteins, which has already been well documented in the literature.^2^ Signals in the 1047–1000 cm^–1^ region arise from C–O and C–O–C stretching of polysaccharides and the glycerol backbone of lipids. The bands between 720 and 660 cm^–1^ reinforcing the presence of unsaturated fatty acids in the almond as well.

Overall, the comparison between almond and oil spectra reveals the expected loss of protein- and carbohydrate-associated bands after extraction, while the lipid bands become more pronounced in the oil. The absence of shifts in the ν­(C=O) and ν­(C–O–C) regions indicates that the extraction did not disrupt the triacylglycerol structure. Although divalent minerals, such as Mg and Zn, may interact with unsaturated fatty acids, these interactions cannot be resolved by FTIR due to the dominance of the ester carbonyl band and extensive overlap in the 1700–1400 cm^–1^ region. Importantly, no bands associated with oxidative degradation were detected, supporting the chemical stability of both matrices prior to thermal analysis.

### 3.3 Thermal Stability

The thermal stability of baru oil and almond was evaluated by thermogravimetric analysis (TGA) and derivative thermogravimetric analysis (DTGA) (Figure 2), providing complementary evidence of how matrix composition and mineral distribution influence degradation behavior. Baru oil exhibited a single, well-defined thermal decomposition event, with an onset temperature (T_onset_) of 394.9 °C and a total mass loss of 98.4% (Figure 2a). This onset is higher than those reported for cucurbita seed (336.1 °C).^21^ The DTGA curve (Figure 2b) showed a maximum degradation rate at 430.1 °C (14.8%/min), values markedly higher than those reported for soybean oil (307 °C), olive oil (316 °C), mustard oil (334 °C), and karanja oil (202 °C),^22^ suggesting its better thermal stability.

**Figure 2:**
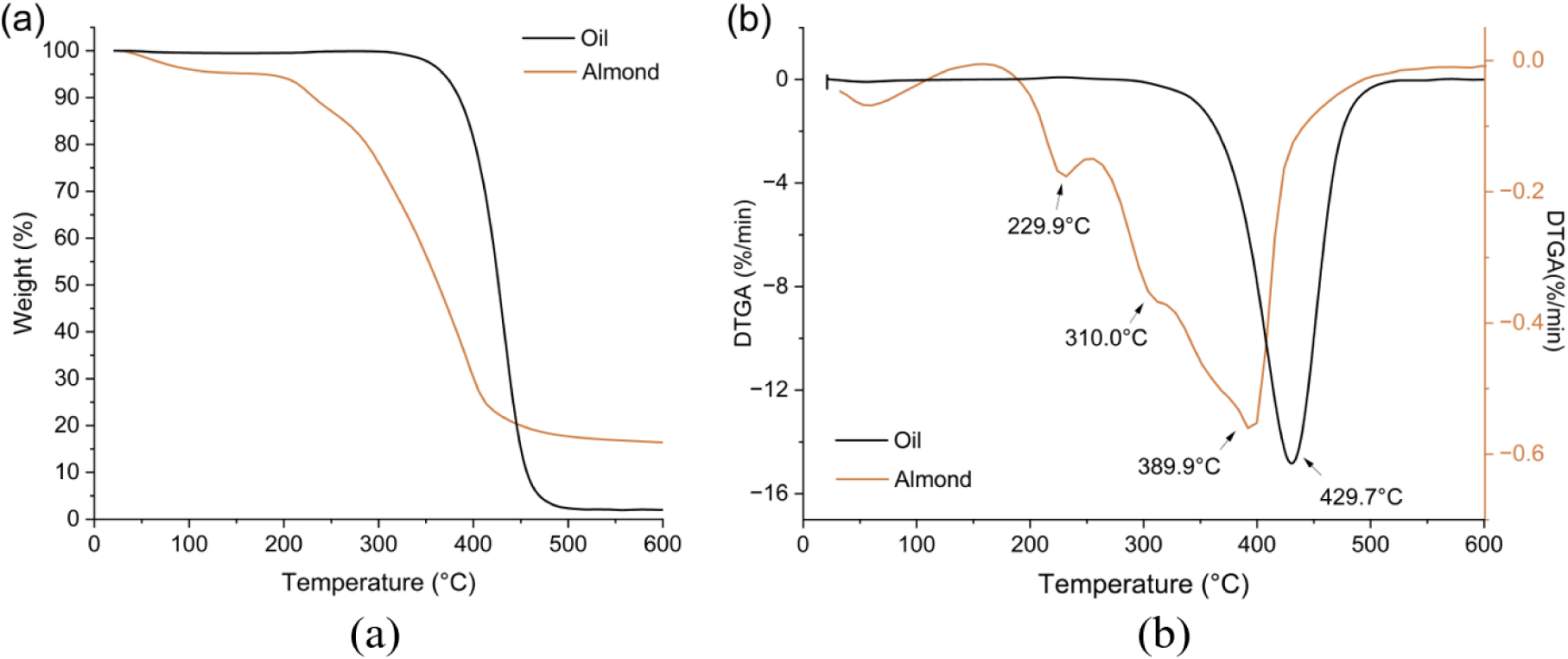
TGA (a) and DTG (b) curves of baru oil and almond.

The thermal resistance of vegetable oils is strongly influenced by their fatty acid compositions and chemical structures. Oils richer in monounsaturated fatty acids generally exhibited higher stability,^23^ whereas polyunsaturated-rich oils degrade earlier. In this context, the high thermal stability of baru oil is consistent with its fatty acid profile, dominated by oleic acid (51.63%), followed by linoleic acid (28.47%) and saturated fatty acids (15.70%), including lignoceric acid or tetracosanoic acid (C24:0) (1.80%). Once, this compound was a very long-chain fatty acid found in palm oil, related to the high thermal and oxidative stability that makes palm oil used as a frying oil globally.^24^

In contrast to the single-step degradation of the oil, the baru almond exhibited a multistage thermal profile, reflecting its heterogeneous composition of lipids, proteins, and carbohydrates.^2^ The almond showed a small low-temperature mass loss attributable to bound moisture, followed by a major degradation event between 230 and 350 °C, associated with the pyrolysis of polysaccharides and proteins. A second, broader degradation stage occurred between 350 and 450 °C, corresponding to lipid decomposition.

### 3.4 Physicochemical Analysis

Table 2 shows the results for the quality parameters of the oil obtained from baru almond. The acidity index (0.37 ± 0.13 mg KOH/g) complied with the limits established by the Codex Alimentarius,^25^ which specify a maximum acidity of 4.0 mg KOH/g for virgin oil and 0.6 mg KOH/g for refined oils. This value is comparable to that reported by Pineli et al.^26^ for cold-pressed baru oil subjected to mild refining steps (filtration, degumming, and bleaching), which showed acidity values between 0.44 ± 0.01 and 0.92 ± 0.02 mg KOH/g after storage under controlled conditions. These results indicate that baru oil presents good quality, with low hydrolytic rancidity and minimal formation of free fatty acids.

Acidity and peroxide indexes are the most used oil quality indexes and are a reflection of processing conditions, such as extraction, refining processes, as well as storage.^27^ The peroxide index is an indication of oil deterioration measured by the hydroperoxide concentration, which is the product of the oxidative processes. The peroxide index obtained (6.45 ± 0.26 mEq/kg) was also below Codex limits^25^ (15 mEq/kg for virgin oils and 10 mEq/kg for refined oils), suggesting low levels of primary oxidation. The lower formation of oxidation compounds may be associated with the presence of natural antioxidants in baru, such as phenolic compounds,^28^ that contribute to delaying the degradation process of the oil obtained. The peroxide index was lower than those reported for baru oil (13.20 ± 1.39 mEq/kg–14.23 ± 1.19 mEq/kg).^29^ The reduced formation of oxidative compounds is also related to the limited migration of transition metals into the oil, as demonstrated by the mineral profile.

The saponification index of baru oil (285.61 ± 2.90 mg KOH/g) was higher than values reported in previous studies (209.59 mg KOH/g and 188.2 mg KOH/g).^30^ Such differences may arise from variations in extraction conditions or environmental factors affecting fruit maturation. The high saponification value suggests a higher proportion of medium-molecular-weight fatty acids, since a higher potassium hydroxide concentration is necessary to neutralize the high level of carboxylic groups.

The refractive index and relative density of vegetable oils are also essential analyses to identify and characterize vegetable oils. Baru oil at 40 °C showed a 1.48 ± 0.02, similar to those reported by other authors for baru oil of 1.47.^31^ According to Davis et al.,^32^ high refractive index values are expected for unsaturated fatty acids.

The relative density of vegetable oils is dependent on their fatty acids and other minor components, as well as on the involved temperature.^33^ Baru oil showed in the current research a relative density of 0.931 ± 0.002 g/mL, values similar to those previously reported for baru oil (0.912 g/mL).^31^ The density of vegetable oils is directly proportional to the degree of unsaturation.^34^ The result is also in accordance with the density values reported for other vegetable oils such as macauba (0.917 g/mL),^33^ soybean (0.917 g/mL), and rapeseed (0.912 g/mL) oils.^35^ Besides, the density of vegetable oils can also be influenced by the fatty acid composition, the presence of other minor components, and the involved temperature.^33^

Taken together, the physicochemical results indicate that baru oil exhibits good oxidative quality, a favorable fatty acid profile, and structural integrity. When integrated with FTIR and thermal analyses, these findings highlight an important connection between mineral partitioning and oil stability: the retention of transition metals in the almond, rather than in the oil, may contribute to the superior oxidative and thermal stability of the oil, as evidenced by its low peroxide index and high T_onset_ value in TGA. These combined results reinforce the technological potential of baru oil for applications that require stability during processing and storage.

### 3.5 Fatty Acids Composition

The fatty acid profile of the baru oil is presented in Table 3. Oleic acid (C18:1) and linoleic acid (C18:2) were the predominant components, accounting for 51.62% and 28.47%, respectively. Overall, the fatty acid distribution followed the order: C18:1 > C18:2 > C16:0 > C18:0 > C18:3 > C24:0 > C20:0 > C22:0 > C16:1 > C17:0 > C14:0.

**Table 3:** Fatty Acids Composition of Baru Oil

Number of carbons	Fatty acids	Fatty acids (%)
C14:0	Myristic acid	0.04
C16:0	Palmitic acid	7.58
C16:1	Palmitoleic acid	0.12
C17:0	Marginic acid	0.04
C18:0	Stearic acid	4.86
C18:1 n9c	Oleic acid	51.62
C18:2 n6c	Linoleic acid	28.47
C18:3 n3	Gamma-linoleic acid	4.09
C20:0	Arachidic acid	0.89
C22:0	Behenic acid	0.49
C24:0	Lignoseric acid	1.80
Saturated total	-	15.70
Unsaturated total	-	84.30

The dominance of oleic and linoleic acids, around 80%, was slightly higher than values reported for mechanically extracted oil (74.76%)^26^ and for the supercritical fluid extraction method (77.94%).^29^ The results indicate that the heat-solvent extraction method applied in this study did not lead to measurable degradation of unsaturated fatty acids. This interpretation is consistent with the low peroxide value observed and with the FTIR spectrum, which showed no shifts in the ester carbonyl region, indicative of oxidative deterioration.

The fatty acid profile reinforces the physicochemical behavior observed for baru oil, particularly its high oleic acid content, which is consistent with the refractive index, density, and saponification values. This monounsaturated-rich composition contributes to the oxidative and thermal stability, as confirmed by FTIR and TGA. When integrated with the mineral profile, these findings become even more meaningful: although the almond contains high levels of transition metals, their limited migration into the oil reduces the susceptibility of its unsaturated fatty acids to oxidative degradation.

### 3.6 Rheological Characterization

The flow curves (Supporting Information–Figure S1A) showed a linear increase in shear stress with shear rate from 10 to 45 °C, indicating predominantly Newtonian behavior at processing-relevant regions. No significant differences (*p* > 0.05) were observed between the curves obtained at 10–15 °C and 15–25 °C, suggesting similar flow resistance within these intervals. As temperature increased, shear stress and apparent viscosity decreased (Supporting Information–Figure S1B), reflecting the enhanced molecular mobility of triacylglycerols.^34^ This trend has been widely reported for vegetable oils, and it is attributed to reduced intermolecular interactions within the lipid matrix at higher temperatures.^36^

The Power law model (Table 4) best fitted the data (R^2^ > 0.99), although deviations in the flow behavior index (*n*) at low temperatures were minimal and restricted to low shear rates. At shear rates above 10 s^–1^, baru oil behaved essentially as a Newtonian fluid across all temperatures, in agreement with reports for other unstructured vegetable oils.^37^

**Table 4:** Rheological Parameters of Baru Oil

		**Temperature (°C)**				
Model	Parameters	10	15	25	35	45
**Power Law**	***K***	147.289	49.398	27.448	18.982	12.392
	***n***	0.925	1.157	1.174	1.154	1.153
	**R** ^**2**^	0.999	0.997	0.995	0.995	0.995
	**Error**	0.001	0.001	0.002	0.001	0.001
**Herschel–Bulkley**	**τ_0_**	0	0.770	0.008	0.008	0.008
	***K***	0.135	0.025	0.057	0.039	0.028
	***n***	0.950	1.283	1.00	0.998	0.990
	**R** ^**2**^	0.999	0.993	0.999	0.999	0.999
	**Error**	1.983	1.517	1.360	1.980	1.810
**Bingham**	**η_pl_**	0.078	0.013	0.001	0.016	0.025
	***K***	0.112	0.090	0.058	0.039	0.027
	***n***	1.00	1.00	1.00	1.00	1.00
	**R** ^**2**^	0.999	0.999	0.999	0.999	0.999
	**Error**	2.010	1.993	1.980	2.580	2.360

η_pl_: plastic viscosity; *K*: consistency index (Pa·s^n^); *n*: flow behavior index (dimensionless); R^2^: correlation coefficient; and τ_0_: initial shear stress. The equations corresponding to the applied rheological models are provided in the Supporting Information.

Temperature dependence of viscosity was evaluated by the Arrhenius model (Supporting Information–Table S1). The activation energy of flow (*E*_a_) of 31.08 kJ/mol is consistent with values reported for other vegetable oils, such as canola (31.7 kJ/mol),^38^ buriti (30.2 kJ/mol),^39^ and rapeseed (30.1 kJ/mol)^40^ oils. The result indicates a moderate temperature sensitivity of viscosity within the evaluated temperature range (10–45 °C). As *E*_a_ is associated with molecular mobility and intermolecular interactions,^40^ the observed behavior suggests typical flow characteristics for oils rich in oleic and linoleic acids.

Despite this sensitivity, baru oil displayed excellent oxidative and thermal stability, as demonstrated by its high T_onset_ in TGA and the absence of carbonyl shifts in FTIR. This stability likely arises from the composition dominated by oleic acid and from the low levels of transition metals present in the oil, which reduce catalytic oxidative degradation during heating.

In conclusion, this study provides the first integrated assessment of the mineral composition, structural features, and techno-functional characterization of baru almond and its extracted oil. The almond exhibited high levels of essential minerals, such as Mn, Fe, Cu, Zn, and Ca, whereas a limited transfer of transition metals to the oil phase was observed. The oil was characterized by a predominance of oleic and linoleic acids along with the presence of very-long-chain saturated fatty acids such as lignoceric acid. Thermal analysis indicated high onset degradation temperatures, and FTIR spectra did not show bands associated with oxidation, supporting the observed thermal and oxidative stability. Rheological analysis indicated predominantly Newtonian behavior at shear rates relevant to processing conditions. These findings address the proposed objectives by establishing the compositional and physicochemical profiles of baru oil. Further studies are required to evaluate extraction scale-up and to validate functional performance under industrial conditions. Together, these results may contribute to the valorization of Cerrado biodiversity and support its sustainable utilization.

## Supplementary Material



## Notes

Rheological models used for data fitting (Newton, Ostwald–de Waele, Bingham, and Casson models), rheological parameters obtained by Arrhenius model, flow curves and apparent viscosity of baru oil at different temperatures, and the viscosity–temperature relationship of baru oil (PDF)
