# Supplementary material for: Integrated Mineral Profiling and Techno-Functional Characterization of Baru (Dipteryx alata Vogel) Oil and Almond
Source: ACS Omega. 2026 Jun 4;11(23):33655–62. doi: 10.1021/acsomega.5c13208 (PMC13280906; doi:10.1021/acsomega.5c13208)
Supplement: Supplementary file 1 [file ao5c13208_si_001.pdf]

Integrated mineral profiling and techno-functional characterization of baru (*Dipteryx alata* Vogel) oil and almond

Pamela F. M. Pereira[a], *Ilma Marques Gomes*[b], *Renato Queiroz Assis*[b], *Gabriel Bezerra Cardoso*[b], *Renata Lázara de Araújo*[b], *Danilo Hiroshi Konda*[b], *Paula Becker Pertuzatti*[b*]

[a]Division of Glycoscience, School of Biotechnology, KTH Royal Institute of Technology, AlbaNova University Centre, SE–106 91 Stockholm, Sweden.

[b]Institute of Exact and Earth Sciences, Federal University of Mato Grosso, Barra do Garças, 78600-000, Brazil.

Rheological models:

Newton:

$$\tau = \eta\dot{\gamma} \quad (1)$$

where η is Newtonian viscosity in Pa.s and $\dot{\gamma}$ is the shear rate in s^{-1}.

Ostwald-de-Waele (Power law):

$$\tau = K.\dot{\gamma}^{n} \quad (2)$$

where K is the consistency index in Pa.s^n; $\dot{\gamma}$ is the shear rate in s^{-1}; and n is the behavior index (dimensionless).

Bingham:

$$\eta = \eta_p + \frac{\tau_0}{\dot{\gamma}} \quad (3)$$

where η_p is the Bingham plastic viscosity in Pa.s; τ_o initial shear stress (Pa); and $\dot{\gamma}$ is the shear rate in s^{-1}.

Casson:

$$\eta = \sqrt[n]{\frac{\tau_0}{\dot{\gamma}} + \left(\eta_p\right)^n} \quad (4)$$

where η_p is the Casson plastic viscosity in $(Pa.s)^n$; τ_o initial shear stress (Pa); and $\dot{\gamma}$ is the shear rate in s^{-1}; n behavior index (dimensionless).

Table S1 – Rheological parameters of baru oil in at different temperatures by Arrehnius model.

**E_a (kJ.mol^{-1})**	**η_0 (mPa.s)**	**(R^2)**
31.06	2.18x10^{-4}	0,997

E_a: activation energy (kJ.mol^{-1}); η_0: adjustment parameter (dimensionless) and R^2: correlation coefficient.

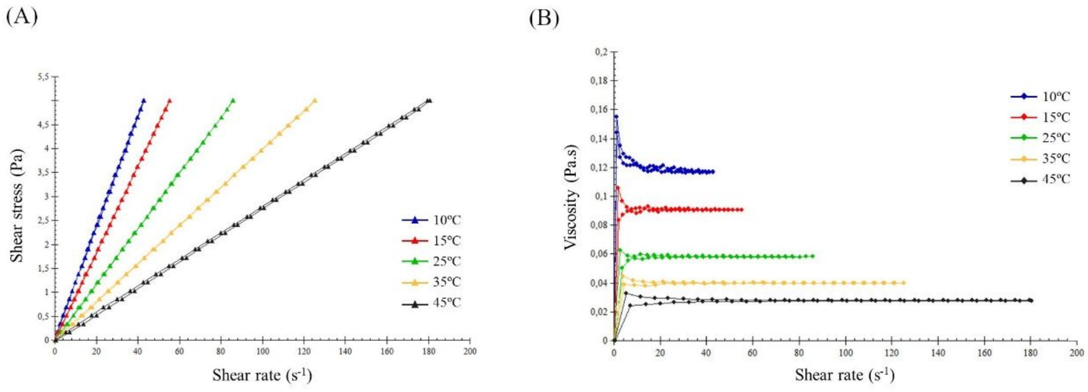

Figure S1 – Flow curves (A) and apparent viscosity (B) of baru oil at different temperatures of 10ºC, 15ºC, 25ºC, 35ºC, and 45ºC.

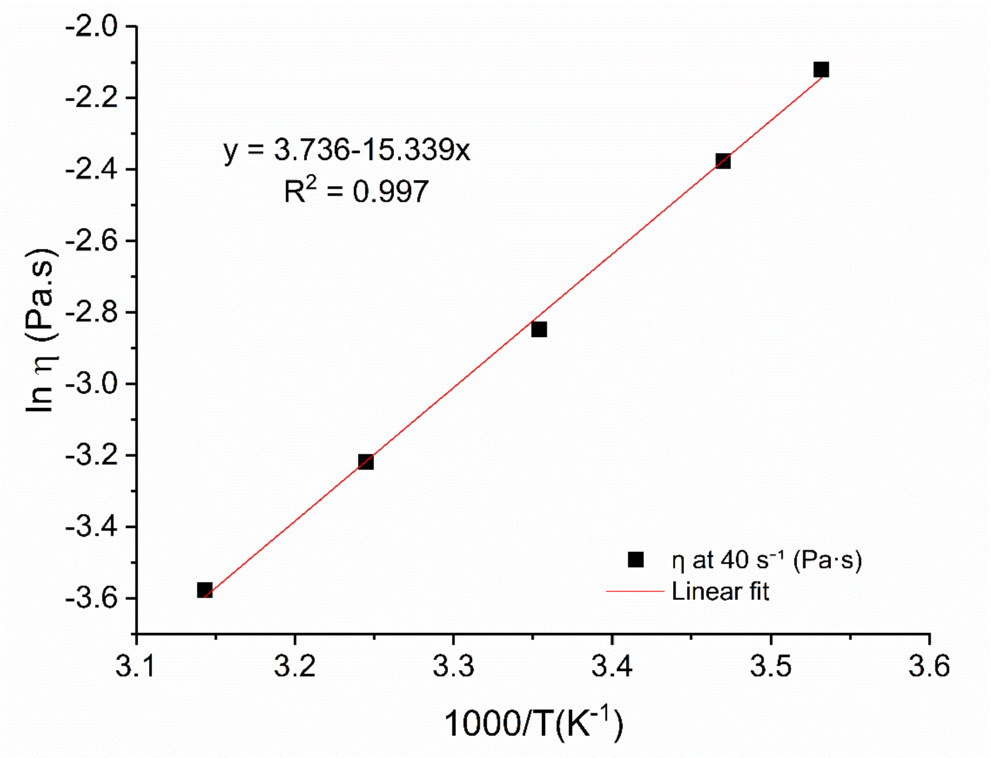

Figure S2. Viscosity–temperature relationship of baru oil
